# Albuminuria, glomerular filtration rate, and fracture among U.S. adults with and without type 2 diabetes

**DOI:** 10.1093/jbmrpl/ziag166

**Published:** 2026-10-09

**Authors:** Simon Hsu, Leila R Zelnick, Ian H de Boer, Mishaela R Rubin, Thomas L Nickolas

**Affiliations:** Division of Nephrology and Kidney Research Institute, Department of Medicine, University of Washington, Seattle, WA 98104, United States; Division of Nephrology and Kidney Research Institute, Department of Medicine, University of Washington, Seattle, WA 98104, United States; Division of Nephrology and Kidney Research Institute, Department of Medicine, University of Washington, Seattle, WA 98104, United States; Metabolic Bone Disease Unit, Department of Medicine, Columbia University, New York, NY 10032, United States; Division of Bone and Mineral Diseases, Department of Medicine, Washington University in St. Louis, St. Louis, MO 63110, United States

**Keywords:** DXA, fracture risk assessment, other diseases and disorders of/related to bone, other disorders of calcium/phosphate metabolism, other systems biology—bone interactions

## Abstract

CKD and type 2 diabetes (T2D) are established risk factors for fracture. Albuminuria and reduced estimated glomerular filtration rate (eGFR), the most commonly used measures to diagnose CKD, may confer fracture risk through distinct mechanisms. Clarifying the associations of albuminuria, reduced eGFR, and T2D status with fracture risk and potential interactions between these exposures may improve fracture risk assessment. We performed a cross-sectional study of 20 034 participants 50 yr or older in the National Health and Nutrition Examination Survey (NHANES) from 1999 to 2020. Albuminuria was defined by spot urine albumin-to-creatinine ratio ≥30 mg/g; reduced eGFR was defined as <60 mL/min/1.73 m^2^. Outcomes were prevalent hip and any fracture—the composite of any hip, wrist, or spine fracture—ascertained using self-report of physician diagnosis. Mean age was 63 yr; 53% were female, 9% were non-Hispanic Black, and 5% were Mexican–American. Participants reported 417 hip and 2703 any fractures. After adjusting for demographic and clinical covariates, including osteoporosis diagnosis and treatment, albuminuria was associated with increased odds of hip fracture (OR 1.59, 95% CI: 1.13-2.22), with no significant interaction by eGFR or T2D status. Neither reduced eGFR (OR 1.02; 95% CI: 0.73-1.44) nor T2D status (OR 0.94; 95% CI: 0.63-1.39) was associated with higher odds of hip fracture. Albuminuria, reduced eGFR, and T2D were not significantly associated with any fracture. In a subset of 7005 participants with BMD measurements, additional adjustment for FN BMD partially attenuated the association of albuminuria with hip fracture (OR 1.35; 95% CI: 0.72-2.53). In conclusion, albuminuria is associated with hip fracture in adults with and without T2D, independent of eGFR.

## Introduction

CKD is a major public health problem and confers an increased risk of fracture.^1^^,^^2^ Altered mineral metabolism and bone turnover characterize the systemic CKD-mineral and bone disorder (CKD-MBD), which is highly prevalent across all stages of CKD and nearly universal in advanced disease.^3^ These impairments occur early in the course of CKD and lead to defects in BMD, skeletal fragility, and elevated fracture risk.

CKD is most commonly defined and staged using estimated glomerular filtration rate (eGFR) and albuminuria.^4^ Most studies of fracture risk in CKD populations have assessed eGFR as a risk factor^5–11^; however, multiple studies suggest that reduced eGFR and albuminuria reflect distinct pathophysiologic mechanisms of kidney injury and may therefore have unique clinical consequences. For example, albuminuria confers excess cardiovascular and mortality risk beyond what is attributable to reduced eGFR alone,^12^^,^^13^ and each of the two markers associates with distinct complications of CKD.^14^ Bone is highly vascularized and disruptions in its microcirculation may lead to bone loss.^15^^,^^16^ Understanding whether albuminuria, a marker of microvascular disease, is an independent risk factor for fracture has important implications for fracture risk assessment and management.

Type 2 diabetes (T2D) is the leading cause of CKD worldwide and in the United States.^17^ Type 2 diabetes has been linked to higher fracture risk though mechanisms independent of kidney function, including fall-related mechanisms, such as neuropathy and retinopathy, as well as changes to bone composition by advanced glycation end products.^18^ Type 2 diabetes may modify the CKD-fracture relationship, but this has not been well characterized.

In this study, we assessed the associations of albuminuria, reduced eGFR, and T2D status with fracture risk and examined potential interactions between these exposures.

## Materials and methods

### Study population

The National Health and Nutrition Examination Survey (NHANES) is a series of nationally representative cross-sectional studies designed to assess the health and nutritional status of adults and children in the United States. Participants undergo both physical examinations and interviews consisting of demographic, socioeconomic, dietary, and health-related questions. NHANES uses a probability sampling design, which intentionally oversamples participants of Black race, Hispanic ethnicity, or both.

For this study, we used data from eight NHANES cycles: 1999-2000, 2001-2002, 2003-2004, 2005-2006, 2007-2008, 2009-2010, 2013-2014, 2017-March 2020. Data collection details have been described elsewhere.^19^^,^^20^ NHANES cycles 2011-2012 and 2015-2016 did not include an osteoporosis questionnaire, which was used to ascertain fractures, and were therefore excluded. We included all NHANES participants 50 yr or older who underwent an NHANES mobile examination center health examination, responded to the osteoporosis questionnaire, and had available data on prescription medication use, hemoglobin A1c (HbA1c), serum creatinine concentration, and urine albumin and creatinine concentrations (Figure S1). We excluded participants with presumed type 1 diabetes, defined as participants with a diagnosis of diabetes before 30 yr of age with use of insulin within 2 yr of diabetes diagnosis and current insulin use.^21^ We also excluded participants with kidney failure on maintenance dialysis.

### Estimated GFR and albuminuria

Estimated GFR was calculated using the 2021 CKD-EPI creatinine equation.^22^ Albuminuria was defined as a urine albumin-to-creatinine ratio (ACR) of *>*30 mg/g. Serum and urine creatinine concentrations were measured using the kinetic Jaffe rate reaction method, whereas urine albumin concentration was ascertained via a solid-phase fluorescent immunoassay from a single voided urine sample.

### Definition of T2D

Type 2 diabetes (T2D) was defined as the use of insulin or other glucose-lowering medications, a HbA1c *>*6.5%, or both, in persons diagnosed with diabetes at 30 yr of age or older without the continuous use of insulin.^23^ Hemoglobin A1c was measured using high performance liquid chromatography (coefficients of variation <2.0%) and was calibrated using a previously developed equation.^24^

### Fracture ascertainment

Fracture history, osteoporosis diagnosis and treatment history, and a history of daily glucocorticoid use were ascertained using the NHANES osteoporosis questionnaire (OSQ) administered by trained interviewers. Fractures were defined by self-report of physician diagnosis, where any fracture was defined as a physician-diagnosed fracture of hip, wrist, or spine. Osteoporosis diagnosis was defined by self-report of physician diagnosis, and treatment was defined by self-report of prescription osteoporosis medications. Daily glucocorticoid use was defined as self-reported use of prednisone or cortisone pills daily for one month or longer.

### Other clinical characteristics

Age, sex, race, ethnicity, duration of diabetes, and smoking status were assessed by questionnaires administered by trained interviewers. Systolic and diastolic blood pressures (BP) were reported as the mean of three consecutive seated BP readings. 25OHD concentrations were measured using liquid chromatography–tandem mass spectrometry (LC-MS/MS) or, for cycles prior to 2007, standardized to LC-MS/MS equivalent values using NHANES-recommended equations.^25^ High sensitivity C-reactive protein concentrations were measured using latex-enhanced nephelometric or two-reagent immunoturbidimetric methods depending on the survey cycle as described in the NHANES laboratory procedure manuals. Femoral neck BMD was measured by DXA across 5 of the 8 NHANES cycles included in this study.

### Statistical analysis

All analyses were performed using R, version 4.4.3 (R Foundation for Statistical Computing) and Stata, version 15.1 (StataCorp), using Stata’s *svy* commands to incorporate NHANES recommendations for weighting to account for nonresponse bias and the sampling design.^19^ Two-sided *p*-values <.05 were considered statistically significant. Complete information on age, sex, race and ethnicity, eGFR and urine ACR, diabetes status, and osteoporosis and fracture history was available for the final analytic sample.

We examined temporal trends of unadjusted hip and any fracture by albuminuria, eGFR, and T2D status across NHANES cycles. We used logistic regression to test independent associations of albuminuria, eGFR <60 mL/min/1.73 m^2^, and T2D status with prevalent fractures. For each of the two fracture outcomes—hip and any fracture—we fitted 2 models: model 1 adjusted for age, age squared, sex, race and ethnicity, and NHANES cycle, and model 2 additionally adjusted for smoking status, history of osteoporosis diagnosis and treatment, history of daily glucocorticoid use, and body mass index. We also used logistic regression to test associations of combinations of T2D status, eGFR, and albuminuria with prevalent fractures, using the same covariate adjustments as model 1 and model 2. Participants were categorized into eight mutually exclusive groups defined by the presence or absence of albuminuria, eGFR above or below 60 mL/min/1.73 m^2^, and T2D status, with those having no albuminuria, eGFR *>*60, and no T2D as the reference group. We further examined associations of albuminuria with fracture outcomes stratified by T2D status and eGFR. Interactions were tested using the Wald test of the product terms albuminuria × T2D and albuminuria × eGFR (*>*60 vs <60). In separate models, we additionally adjusted for FN BMD (restricted to 7005 participants with available DXA measurements) and for hs-CRP (restricted to 17 519 participants with available measurements) to assess whether BMD or inflammation might explain albuminuria-fracture associations. We tested for interaction between albuminuria and BMD using the Wald test of their product term, with additional adjustment for eGFR. Lastly, we examined the unadjusted prevalence of hip and any fracture by albuminuria, eGFR, and T2D status, stratified by FN T-score categories in the subset of participants with DXA data.

## Results

### Participant characteristics

The study population consisted of 20 034 adults: 3651 with albuminuria and 16 383 without. Weighted mean (SD) age was 63 (8.5) yr, with 53% females, 9% self-identifying as non-Hispanic Black, and 5% as Mexican–American (Table 1). Overall, 18% had T2D, and 12% had eGFR <60 mL/min/1.73 m^2^ with a weighted mean (SD) eGFR of 83 (17) mL/min/1.73 m^2^ and urine ACR of 46 (279) mg/g. Compared with participants without albuminuria, those with albuminuria were older, more likely to be non-White race, and more likely to have hypertension and lower eGFR. Mean 25OHD concentrations were marginally lower in the albuminuria group (26.2 vs 29.0 ng/mL). Participants with albuminuria also had higher HbA1c, were more likely to have T2D, and among those with T2D, had a longer duration of diabetes.

**Table 1:** Characteristics of participants at NHANES enrollment, among those *>*50 yr of age.

	Overall (*N* = 20 034)	No albuminuria (*N* = 16 383)	Albuminuria (*N* = 3651)
**Weighted percentage of participants (95% CI)**		86% (85%-87%)	14% (13%-15%)
**Age (yr), weighted mean (SD)**	63 (8.5)	63 (8.0)	67 (10.3)
**Female, weighted %**	53%	53%	52%
**Race and ethnicity, weighted %**			
**Non-Hispanic White**	77%	78%	69%
**Non-Hispanic Black**	9%	8%	13%
**Mexican-American**	5%	4%	6%
**Other^a^**	10%	10%	12%
**Type 2 diabetes, weighted %**	18%	15%	40%
**Duration of diabetes (yr), weighted mean (SD)**^**b**^	3.8 (11.9)	2.9 (10.2)	9.5 (20.1)
**Hypertension, weighted %**	74%	71%	90%
**Osteoporosis, weighted %**	11%	11%	13%
**Current smoker, weighted %**	16%	15%	18%
**History of osteoporosis treatment, weighted %**	7%	7%	8%
**History of daily glucocorticoid use, weighted %**	5%	5%	5%
**BMI (kg/m** ^**2**^ **), weighted mean (SD)**	29.2 (5.7)	29.0 (5.4)	29.9 (7.1)
**Systolic BP (mmHg), weighted mean (SD)**	130 (18)	129 (16)	141 (25)
**Diastolic BP (mmHg), weighted mean (SD)**	71 (12)	71 (11)	71 (17)
**Hemoglobin A1c (%), weighted mean (SD)**	5.8 (0.9)	5.8 (0.7)	6.4 (1.6)
**eGFR (mL/min per 1.73 m** ^**2**^ **), weighted mean (SD)**	83 (17)	84 (15)	76 (24)
**eGFR <60 mL/min per 1.73 m**^**2**^**, weighted %**	12%	9%	26%
**Urine albumin-to-creatinine (mg/g), weighted mean (SD)**	46 (279)	9 (5)	277 (816)
**Calcium (mg/dL), weighted mean (SD)**	9.4 (0.3)	9.4 (0.3)	9.4 (0.5)
**Phosphate (mg/dL), weighted mean (SD)**	3.7 (0.5)	3.7 (0.5)	3.7 (0.6)
**25OHD (ng/mL), weighted mean (SD)**	28.6 (9.3)	29.0 (9.0)	26.2 (10.7)

a Includes other Hispanic, non-Hispanic Asian, and multiracial participants according to NHANES classifications.

b Among those with type 2 diabetes.

Abbreviations: BP, blood pressure; eGFR, estimated glomerular filtration rate; NHANES, National Health and Nutrition Examination Survey.

### Fracture prevalence

Participants reported 417 hip and 2703 any fractures. Unadjusted weighted prevalence of hip fracture was higher in participants with albuminuria (3.5% vs 1.6%) and eGFR <60 (3.2% vs 1.7%), but not T2D status (1.8% vs 1.9%; Table 2). When albuminuria, eGFR, and T2D status were examined in combination, unadjusted prevalence of both hip and any fracture was highest among participants with albuminuria and eGFR <60 but without T2D (Figure 1). Unadjusted hip fracture prevalence was higher across most NHANES cycles among participants with albuminuria compared with those without regardless of eGFR category or T2D status (Figures S2 and S3). Hip fracture prevalence appeared to increase among participants with albuminuria and eGFR <60 mL/min/1.73 m^2^ after 2007-2008.

**Table 2:** Independent associations of albuminuria, eGFR, and type 2 diabetes with fractures.

	Hip fracture				Any fracture			
			OR (95% CI)				OR (95% CI)	
	*N* at risk (N events)	Weighted prevalence of fracture, % (95% CI)	Model 1	Model 2	*N* at risk (*N* events)	Weighted prevalence of fracture, % (95% CI)	Model 1	Model 2
**Albuminuria**	3651 (115)	3.5% (2.7%-4.4%)	1.69 (1.25-2.29)^a^	1.59 (1.13-2.22)^a^	3651 (529)	16.2% (14.7%-17.8%)	1.08 (0.96-1.22)	1.03 (0.91-1.16)
**No albuminuria**	16 383 (302)	1.6% (1.3%-1.9%)	Ref	Ref	16 383 (2174)	14.9% (14.1%-15.7%)	Ref	Ref
								
**eGFR <60**	2959 (93)	3.2% (2.5%-4.1%)	1.04 (0.74-1.46)	1.02 (0.73-1.44)	2959 (438)	16.6% (14.8%-18.6%)	1.00 (0.84-1.19)	0.96 (0.80-1.15)
**eGFR *>*60**	17 075 (324)	1.7% (1.4%-2.0%)	Ref	Ref	17 075 (2265)	14.8% (14.0%-15.7%)	Ref	Ref
								
**T2D**	4603 (83)	1.8% (1.3%-2.4%)	0.88 (0.62-1.25)	0.94 (0.63-1.39)	4603 (563)	14.7% (13.1%-16.3%)	0.99 (0.86-1.14)	0.96 (0.83-1.10)
**No T2D**	15 431 (334)	1.9% (1.6%-2.1%)	Ref	Ref	15 431 (2140)	15.1% (14.4%-15.9%)	Ref	Ref

The units of eGFR are mL/min per 1.73 m^2^. Model 1 is adjusted for age, age squared, sex, race and ethnicity, and NHANES cycle. Model 2 additionally adjusts for smoking status, history of osteoporosis diagnosis and treatment, history of daily glucocorticoid use, and BMI.

Abbreviations: eGFR, estimated glomerular filtration rate; OR, odds ratio; T2D, type 2 diabetes.

a *p* < .05.

**Figure 1:**
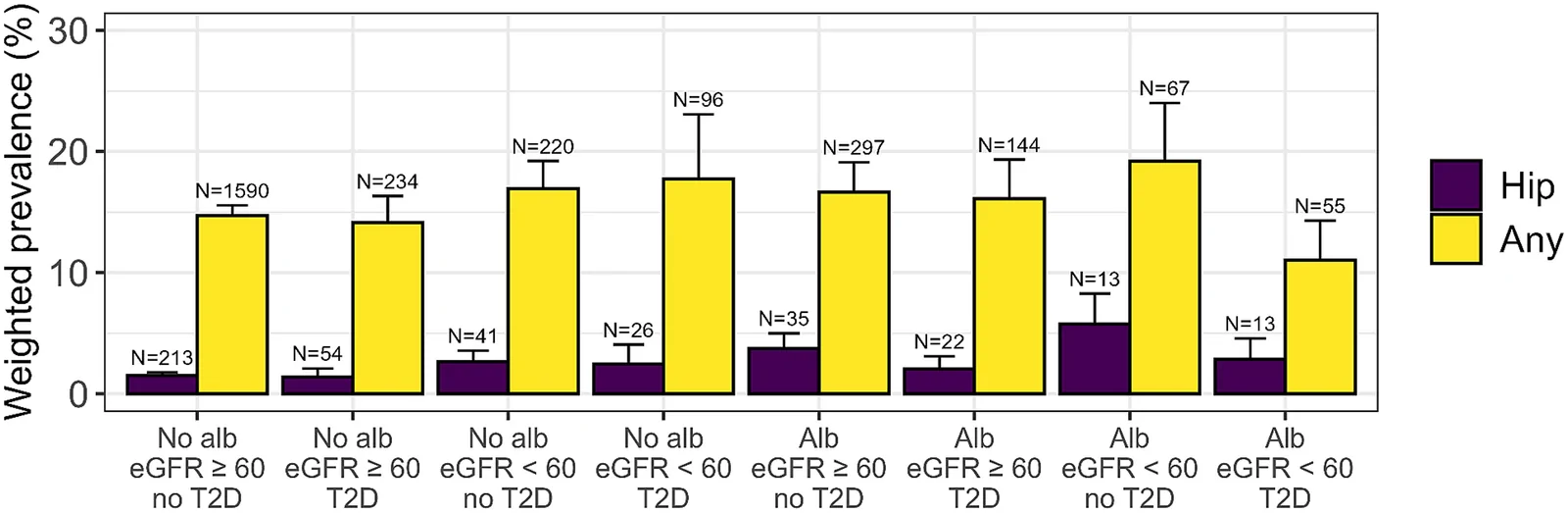
Fracture prevalence in NHANES (1999-2020) by combined albuminuria, eGFR, and type 2 diabetes status. The number of fracture events is presented above each bar. Error bars represent 95% CIs.

### Albuminuria, eGFR, T2D, and fracture risk

After covariate adjustment, participants with albuminuria had significant higher odds of hip fracture compared with those without albuminuria (odds ratio [OR] 1.59; 95% CI: 1.13-2.22; Table 2). Neither eGFR <60 (OR 1.02; 95% CI: 0.73-1.44) nor T2D status (OR 0.94; 95% CI: 0.63-1.39) was associated with higher odds of hip fracture. Albuminuria, eGFR <60, and T2D status were not significantly associated with any fracture. When examining combinations of albuminuria, eGFR, and T2D status, participants with albuminuria, eGFR *>*60 and without T2D had significantly higher odds of hip fracture compared with the reference group (OR 1.77; 95% CI: 1.16-2.72; Table 3). Adjusted ORs for all other groups with albuminuria suggested greater hip fracture risk, but did not reach statistical significance (OR range: 1.30-1.77), while estimates for all groups without albuminuria were close to unity regardless of T2D or eGFR status (OR range: 0.83-1.04). Associations with any fracture were close to unity for all exposure groups.

**Table 3:** Associations of combined albuminuria, eGFR, and type 2 diabetes status with fractures.

	Hip fracture			Any fracture		
		OR (95% CI)			OR (95% CI)	
	*N* at risk (*N* events)	Model 1	Model 2	*N* at risk (*N* events)	Model 1	Model 2
**No albuminuria, eGFR *>*60, no T2D**	11 962 (213)	Ref	Ref	11 962 (1590)	Ref	Ref
**No albuminuria, eGFR *>*60, T2D**	2524 (35)	0.86 (0.51-1.44)	0.93 (0.55-1.59)	2524 (297)	0.98 (0.82-1.17)	0.96 (0.79-1.15)
**No albuminuria, eGFR <60, no T2D**	1409 (41)	0.98 (0.63-1.53)	1.04 (0.66-1.62)	1409 (220)	1.03 (0.84-1.25)	1.01 (0.83-1.23)
**No albuminuria, eGFR <60, T2D**	488 (13)	0.98 (0.47-2.02)	0.83 (0.39-1.78)	488 (67)	1.17 (0.80-1.71)	1.04 (0.70-1.56)
**Albuminuria, eGFR *>*60, no T2D**	1497 (54)	1.94 (1.30-2.90)^a^	1.77 (1.16-2.72)^a^	1497 (234)	1.14 (0.95-1.36)	1.07 (0.89-1.29)
**Albuminuria, eGFR *>*60, T2D**	1092 (22)	1.24 (0.71-2.16)	1.31 (0.71-2.41)	1092 (144)	1.15 (0.90-1.48)	1.09 (0.85-1.41)
**Albuminuria, eGFR <60, no T2D**	563 (26)	1.90 (1.07-3.35)^a^	1.58 (0.83-3.00)	563 (96)	1.16 (0.84-1.60)	1.05 (0.74-1.47)
**Albuminuria, eGFR <60, T2D**	499 (13)	1.15 (0.57-2.33)	1.30 (0.62-2.70)	499 (55)	0.67 (0.47-0.95)	0.65 (0.45-0.94)

The units of eGFR are mL/min per 1.73 m^2^. Model 1 is adjusted for age, age squared, sex, race and ethnicity, and NHANES cycle. Model 2 additionally adjusts for smoking status, history of osteoporosis diagnosis and treatment, history of daily glucocorticoid use, and BMI.

Abbreviations: eGFR, estimated glomerular filtration rate; OR, odds ratio; T2D, type 2 diabetes.

a *p* < .05.

Across all subgroups defined by T2D status and eGFR, associations of albuminuria with hip fracture were in the direction of increased fracture risk (OR range: 1.40-1.77), although not all associations reached statistical significance (Figure 2 and Table S1). Albuminuria was not significantly associated with any fracture in subgroup analyses (Table S2). These associations did not differ by T2D status or eGFR category (*p* for interaction: albuminuria × T2D = 0.56 and 0.62; albuminuria × eGFR = 0.75 and 0.12, for hip and any fracture, respectively).

**Figure 2:**
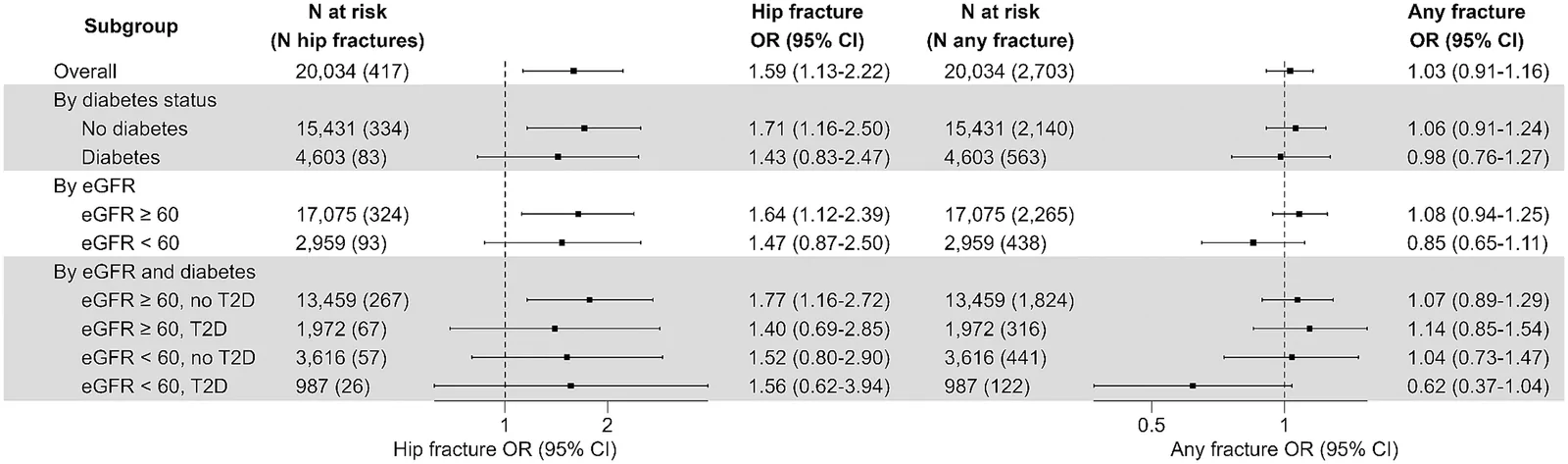
Associations of albuminuria with fractures stratified by type 2 diabetes status and eGFR. The reference group for each stratum is no albuminuria. The units of eGFR are mL/min per 1.73 m^2^. The models are adjusted for age, age squared, sex, race and ethnicity, NHANES cycle, smoking status, history of osteoporosis diagnosis and treatment, history of daily glucocorticoid use, and BMI.

### BMD, inflammation, and fracture risk

In the subset of 7005 participants with DXA measurements, additional adjustment for FN BMD attenuated the association of albuminuria with hip fracture, which was no longer statistically significant, although the point estimate remained in the direction suggesting greater fracture risk (OR 1.35; 95% CI: 0.72-2.53; Table S1). Associations did not differ by BMD (*p* for interaction = .62 and .33 for hip and any fracture, respectively). Most participants with prevalent hip or any fracture had FN T-scores consistent with osteopenia (−2.4 to −1) or osteoporosis (*<*−2.5), except among those with T2D—both with and without albuminuria—in whom any fractures were most prevalent at normal BMD (T-score > −1.0; Figures 3 and 4).

**Figure 3:**
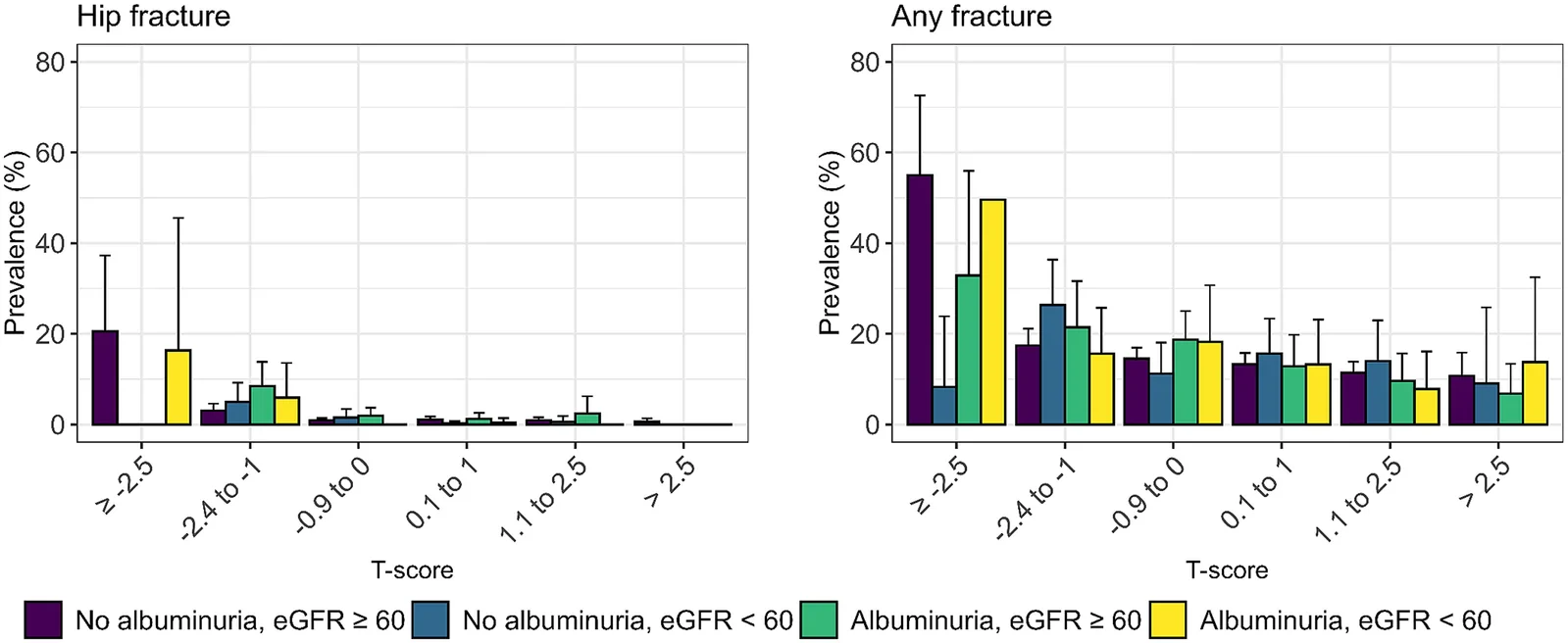
Fracture prevalence by albuminuria and eGFR categories, stratified by FN T-score. The units of eGFR are mL/min per 1.73 m^2^.

**Figure 4:**
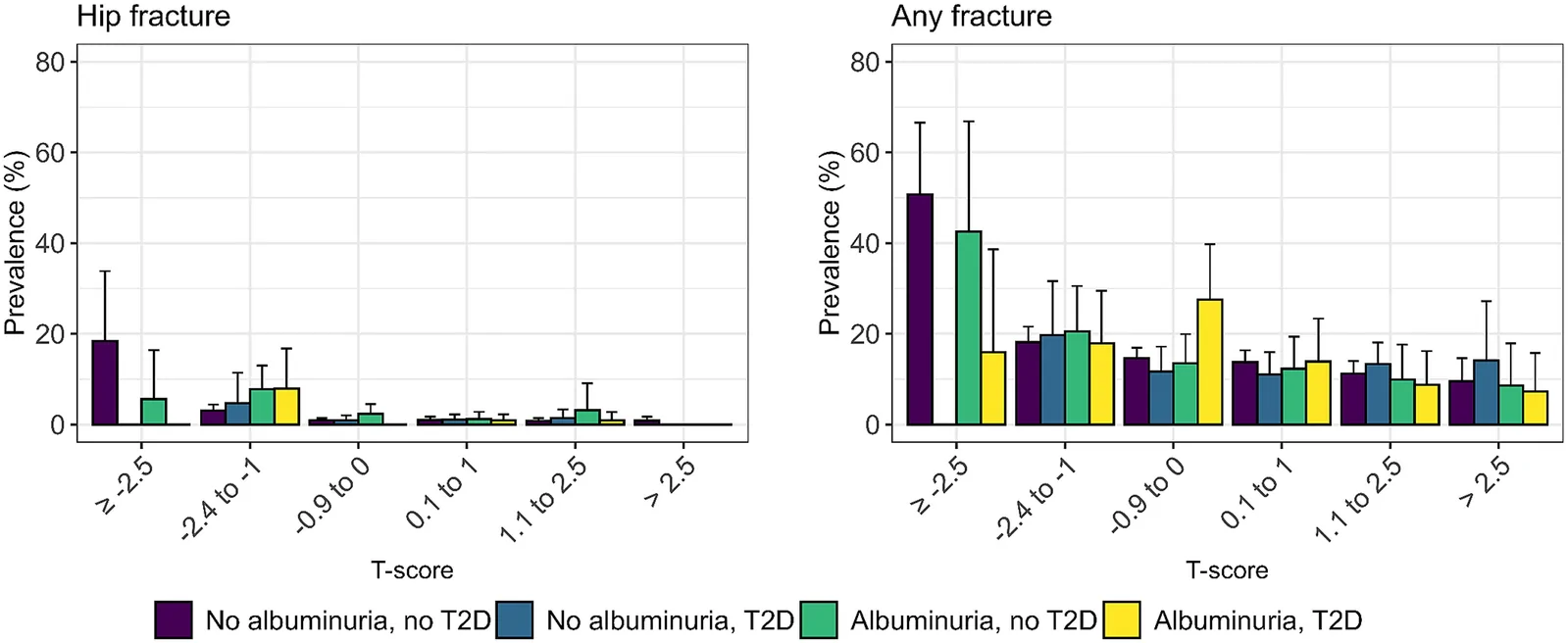
Fracture prevalence by albuminuria and type 2 diabetes categories, stratified by FN T-score.

In the subset of 17 519 participants with hsCRP measurements, additional adjustment for hsCRP did not attenuate the association of albuminuria with hip fracture (OR 1.72; 95% CI: 1.18-2.49; Table S1).

## Discussion

In a large, nationally representative U.S. population of 20 034 adults 50 yr or older spanning a 20-yr period, albuminuria was independently associated with higher odds of hip fracture. Neither eGFR <60 nor T2D was associated with hip fracture or modified the albuminuria-fracture association. In a subset of 7005 participants with DXA measurements, additional adjustment for FN BMD attenuated the association of albuminuria with hip fracture, which was no longer statistically significant, although the estimate remained above unity. Notably, most participants with T2D experienced fractures at normal FN BMD, regardless of albuminuria status.

Observational studies in smaller study populations have tested associations of albuminuria with fractures with mixed findings. Barzilay and colleagues found a weak association in women but not men.^26^ In a subsequent study, they found an association of severe (urine ACR >300 mg/g) but not moderate (urine ACR 30-299 mg/g) albuminuria with hip fractures.^27^ In a male population, Fink and colleagues found that albuminuria associated with faster hip BMD loss, but not incident fractures, though the authors note their study was likely underpowered to detect that association.^28^ Our finding that moderate albuminuria is associated with hip fractures is consistent with those reported in a study of >300 000 South Koreans^29^ and another in >2.7 million residents of Alberta, Canada,^30^ two of the largest community-based population studies on this subject. Our study expands the generalizability of this finding to the racially and ethnically diverse U.S. population. We observed higher unadjusted fracture prevalence in participants with albuminuria and in those with eGFR <60, although after adjusting for demographics, only albuminuria remained significantly associated with hip fracture. This suggests fracture risk associated with reduced eGFR may be largely explained by factors, such as age, while albuminuria confers fracture risk independently of demographic factors. When stratified by T2D status, eGFR category, and combinations of the 2 conditions, not all associations with hip fracture reached statistical significance, likely reflecting reduced power in smaller subgroups with fewer fracture events. Nonetheless, all estimates in these subgroups remained above unity, suggesting albuminuria is an important risk factor for fracture regardless of T2D status or eGFR. Beyond individual-level risk, we note that albuminuria is substantially more prevalent among individuals with T2D than those without.^31^ Consequently, even in the absence of heterogeneity by T2D status, albuminuria may contribute disproportionately to the population burden of fracture among individuals with T2D.

A few mechanisms have been proposed for how albuminuria may increase fracture risk. First, it may do so through reduced BMD. Studies like the one by Barzilay and colleagues link albuminuria with progressive BMD loss,^27^ and our study found that adjusting for FN BMD attenuated the odds of hip fracture, although only partially. Consistent with this, Jørgensen and colleagues similarly found that lower BMD did not fully explain the association between albuminuria and nonvertebral fractures in women, suggesting additional BMD-independent mechanisms.^32^ How albuminuria may reduce BMD is not fully understood. Urinary loss of protein-bound 25OHD may be one pathway,^33^ potentially impairing bone mineralization; however, compensatory vitamin D binding protein synthesis by the liver may attenuate this effect.^34^ In our cohort, participants with albuminuria vs no albuminuria had only marginally lower 25OHD concentrations and were within the normal range, suggesting urinary 25OHD loss is unlikely the primary driver of the albuminuria-fracture relationship. Other mechanisms proposed for how albuminuria may increase fracture risk include inflammation and endothelial dysfunction. Evidence for this largely comes from observational studies associating albuminuria with markers of these pathways, such as c-reactive protein, leukocytosis, and white matter changes on brain imaging,^35^^,^^36^ and studies linking endothelial and perfusion defects with bone loss.^37^^,^^38^ However, whether albuminuria is truly a direct cause of reduced bone strength through these pathways is unclear. A comprehensive analysis of markers of microvascular disease found no significant associations of albuminuria or endothelial markers with BMD.^39^ In our study, additional adjustment for hsCRP did not attenuate the association of albuminuria with fractures, suggesting that systemic inflammation as captured by hsCRP does not explain this relationship. It is also possible that albuminuria and bone fragility are both downstream consequences of a common driver of systemic inflammation and endothelial dysfunction. Future studies are needed to clarify whether albuminuria is causally linked to bone fragility or serves primarily as a marker of systemic vascular injury, and whether interventions that reduce albuminuria are associated with lower fracture risk.

Associations of eGFR—independently of albuminuria—with fracture have been extensively studied with mixed results in non-dialysis populations. Many have found significant associations of reduced eGFR with fracture risk.^5–8^ Others have found positive associations only in certain subgroups, such as women but not men,^9^ more advanced but not early stage CKD,^10^ or with rate of eGFR decline rather than baseline eGFR.^27^ Cooke-Hubley and colleagues report a U-shaped relationship, where participants with eGFR 45-59 had the lowest risk of hip and any fracture, although the authors reflected that individuals with eGFR >90, who had higher fracture risk, may have overestimated eGFRs from low muscle mass and chronic illness.^30^ A large Korean cohort of >550 000 postmenopausal women found that eGFR <45 was associated with greater hip fracture risk only among those with normal BMD, suggesting that severely reduced eGFR may confer BMD-independent fracture risk in select populations.^40^ In contrast with these studies, and consistent with our findings, Elliott and colleagues found no association between eGFR <60 and fracture risk in a population of >1.8 million adults from Alberta, Canada.^11^ These disparate findings likely reflect a combination of factors, including different study populations (eg, age, sex, T2D prevalence, and severity and range of reduced eGFR), variation in adjusted covariates, diminished power in subgroup analyses, and heterogeneity in fracture ascertainment. Unlike non-dialysis CKD, studies consistently associate kidney failure with higher fracture risk^41^; heterogeneity in the underlying processes that drive bone loss across non-dialysis CKD stages, such as CKD-MBD severity, may contribute to the inconsistent eGFR-fracture associations observed in the literature.

Although T2D status did not modify associations of albuminuria with hip fracture, most participants with T2D who experienced fractures had normal FN BMD. The paradox of increased fracture risk despite normal to higher BMD in T2D compared with age-matched healthy peers is well described.^18^^,^^42^^,^^43^ This reflects both increased fall risk, driven by complications like neuropathy, retinopathy, and hypoglycemia, as well as factors hypothesized to compromise bone quality, such as hormonal changes, accumulation of advanced glycation end products, and oxidative stress. A recent review by the International Osteoporosis Foundation Bone and Diabetes Working Group reports that high-resolution imaging studies show greater cortical porosity and thinning despite higher bone density in patients with T2D than healthy controls, which may partially explain the high risk of fracture.^42^ Further mechanistic and longitudinal studies are needed alongside updated risk prediction tools like trabecular bone score and FRAX to improve fracture risk assessment in T2D.

Strengths of this study include the large, nationally representative, racially and ethnically diverse population of U.S. adults captured across two decades; the independent assessment of eGFR and albuminuria as fracture risk factors, stratified by T2D status; the inclusion of osteoporosis diagnosis, treatment, and risk factors as covariates in our models; and the inclusion of BMD and hsCRP in mediation analyses. This study also has important limitations, particularly related to its cross-sectional design. First, we cannot establish temporality between albuminuria and prevalent fracture, and cannot prove that albuminuria is causally linked to fracture. Second, despite adjusting for relevant demographic and clinical covariates, our study remains subject to residual and unmeasured confounding. Third, albuminuria was defined from a single spot urine ACR measurement, which may misclassify participants due to day-to-day variability in urinary albumin excretion. Fourth, most participants had eGFR >60 mL/min/1.73 m^2^, limiting power to detect eGFR-fracture associations in those with reduced eGFR. Fifth, fracture outcomes were self-reported, which cannot distinguish fragility from other causes of fractures such as trauma, and may be subject to recall bias. Finally, because our analysis included only surviving participants and albuminuria is associated with higher mortality,^44^ the highest-risk individuals may have been underrepresented, potentially attenuating the observed associations.

In conclusion, albuminuria is associated with increased odds of hip fracture independently of eGFR and T2D status. Further studies are needed to clarify the pathways through which albuminuria confers fracture risk and how it may be integrated into fracture risk assessment.

## Supplementary Material

NHANES_Albuminuria_Supplement_ziag166(1)

## Data Availability

The datasets analyzed in the current study are publicly available from the National Health and Nutrition Examination Survey (NHANES) repository managed by the Centers for Disease Control and Prevention (CDC) and can be accessed online at https://wwwn.cdc.gov/nchs/nhanes/.

## References

[1] Kim SM, Long J, Montez-Rath M, Leonard M, Chertow GM. Hip fracture in patients with non-dialysis-requiring chronic kidney disease. J Bone Miner Res. 2016;31(10):1803–1809. 10.1002/jbmr.2862 27145189

[2] Hsu S, Bansal N, Denburg M, et al. Risk factors for hip and vertebral fractures in chronic kidney disease: the CRIC study. J Bone Miner Res. 2024;39(4):433–442. 10.1093/jbmr/zjae021 PMC11262146 38477777

[3] Ketteler M, Evenepoel P, Holden RM, et al. Chronic kidney disease-mineral and bone disorder: conclusions from a Kidney Disease: Improving Global Outcomes (KDIGO) Controversies Conference. Kidney Int. 2025;107(3):405–423. 10.1016/j.kint.2024.11.013 39864017

[4] Levin A, Ahmed SB, Carrero JJ, et al. Executive summary of the KDIGO 2024 Clinical Practice Guideline for the Evaluation and Management of Chronic Kidney Disease: known knowns and known unknowns. Kidney Int. 2024;105(4):684–701. 10.1016/j.kint.2023.10.016 38519239

[5] Naylor KL, McArthur E, Leslie WD, et al. The three-year incidence of fracture in chronic kidney disease. Kidney Int. 2014;86(4):810–818. 10.1038/ki.2013.547 24429401

[6] Ensrud KE, Lui LY, Taylor BC, et al. Renal function and risk of hip and vertebral fractures in older women. Arch Intern Med. 2007;167(2):133–139. 10.1001/archinte.167.2.133 17242313

[7] Nickolas TL, McMahon DJ, Shane E. Relationship between moderate to severe kidney disease and hip fracture in the United States. J Am Soc Nephrol. 2006;17(11):3223–3232. 10.1681/ASN.2005111194 17005938

[8] Kim M, Han K, Kwon JK, et al. Bone mineral density and the risk of fracture according to eGFR in postmenopausal women. J Am Soc Nephrol. 2026;37(5):984–994. 10.1681/ASN.0000000960 PMC13143439 41563361

[9] Fried LF, Biggs ML, Shlipak MG, et al. Association of kidney function with incident hip fracture in older adults. J Am Soc Nephrol. 2007;18(1):282–286. 10.1681/ASN.2006050546 17167115

[10] Dooley AC, Weiss NS, Kestenbaum B. Increased risk of hip fracture among men with CKD. Am J Kidney Dis. 2008;51(1):38–44. 10.1053/j.ajkd.2007.08.019 18155531

[11] Elliott MJ, James MT, Quinn RR, et al. Estimated GFR and fracture risk: a population-based study. Clin J Am Soc Nephrol. 2013;8(8):1367–1376. 10.2215/CJN.09130912 PMC3731906 23660179

[12] Hemmelgarn BR, Manns BJ, Lloyd A, et al. Relation between kidney function, proteinuria, and adverse outcomes. Jama. 2010;303(5):423–429. 10.1001/jama.2010.39 20124537

[13] Gansevoort RT, Matsushita K, van der Velde M, et al. Lower estimated GFR and higher albuminuria are associated with adverse kidney outcomes. A collaborative meta-analysis of general and high-risk population cohorts. Kidney Int. 2011;80(1):93–104. 10.1038/ki.2010.531 PMC3959732 21289597

[14] Inker LA, Coresh J, Levey AS, Tonelli M, Muntner P. Estimated GFR, albuminuria, and complications of chronic kidney disease. J Am Soc Nephrol. 2011;22(12):2322–2331. 10.1681/ASN.2010111181 PMC3279937 21965377

[15] Prisby RD . Bone marrow microvasculature. Compr Physiol. 2020;10(3):1009–1046. 10.1002/j.2040-4603.2020.tb00132.x 32941689

[16] Watanabe H, Maishi N, Hoshi-Numahata M, et al. Skeletal-vascular interactions in bone development, homeostasis, and pathological destruction. Int J Mol Sci. 2023;24(13):10912. 10.3390/ijms241310912 PMC10341726 37446097

[17] Koye DN, Magliano DJ, Nelson RG, Pavkov ME. The global epidemiology of diabetes and kidney disease. Adv Chronic Kidney Dis. 2018;25(2):121–132. 10.1053/j.ackd.2017.10.011 PMC11000253 29580576

[18] Moseley KF . Type 2 diabetes and bone fractures. Curr Opin Endocrinol Diabetes Obes. 2012;19(2):128–135. 10.1097/MED.0b013e328350a6e1 PMC4753802 22262002

[19] Centers for Disease Control and Prevention (CDC) . National Center for Health Statistics (NCHS). National Health and Nutrition Examination Survey Data. U.S. Department of Health and Human Services, Centers for Disease Control and Prevention; 2026: https://www.cdc.gov/nchs/nhanes/index.html.

[20] Centers for Disease Control and Prevention (CDC), National Center for Health Statistics (NCHS) . NHANES Analytic Guidance and Brief Overview for the 2017–March 2020 Prepandemic Data Files. National Center for Health Statistics; 2021: https://wwwn.cdc.gov/nchs/nhanes/continuousnhanes/overviewbrief.aspx?Cycle=2017-2020.

[21] Zelnick LR, Weiss NS, Kestenbaum BR, et al. Diabetes and CKD in the United States population, 2009-2014. Clin J Am Soc Nephrol. 2017;12(12):1984–1990. 10.2215/CJN.03700417 PMC5718269 29054846

[22] Inker Lesley A, Eneanya Nwamaka D, Coresh J, et al. New creatinine- and cystatin C–based equations to estimate GFR without race. N Engl J Med. 2021;385(19):1737–1749. 10.1056/NEJMoa2102953 PMC8822996 34554658

[23] Kramer HJ, Nguyen QD, Curhan G, Hsu CY. Renal insufficiency in the absence of albuminuria and retinopathy among adults with type 2 diabetes mellitus. Jama. 2003;289(24):3273–3277. 10.1001/jama.289.24.3273 12824208

[24] Selvin E, Coresh J, Zhu H, Folsom A, Steffes MW. Measurement of HbA1c from stored whole blood samples in the atherosclerosis risk in communities study. J Diabetes. 2010;2(2):118–124. 10.1111/j.1753-0407.2010.00070.x PMC2991637 20923494

[25] Centers for Disease Control and Prevention (CDC), National Center for Health Statistics (NCHS) . Analytical Note for 25-Hydroxyvitamin D Data Analysis Using NHANES III (1988–1994), NHANES 2001–2006, and NHANES 2007–2010 (October 2015). https://wwwn.cdc.gov/nchs/nhanes/vitamind/analyticalnote.aspx.

[26] Barzilay JI, Bůžková P, Chen Z, et al. Albuminuria is associated with hip fracture risk in older adults: the cardiovascular health study. Osteoporos Int. 2013;24(12):2993–3000. 10.1007/s00198-013-2389-3 PMC4504691 23702700

[27] Barzilay JI, Gao P, Clase CM, et al. Albuminuria and rapid loss of GFR and risk of new hip and pelvic fractures. Clin J Am Soc Nephrol. 2013;8(2):233–240. 10.2215/CJN.06640712 PMC3562864 23184565

[28] Fink HA, Vo TN, Langsetmo L, et al. Association of Increased Urinary Albumin with risk of incident clinical fracture and rate of hip bone loss: the osteoporotic fractures in men study. J Bone Miner Res. 2017;32(5):1090–1099. 10.1002/jbmr.3065 PMC5413394 28012217

[29] Kim SH, Yi S-W, Yi J-J, Kim YM, Won YJ. Chronic kidney disease increases the risk of hip fracture: a prospective cohort study in Korean adults. J Bone Miner Res. 2020;35(7):1313–1321. 10.1002/jbmr.3997 32119747

[30] Cooke-Hubley SM, Senior P, Bello AK, Wiebe N, Klarenbach S. Degree of albuminuria is associated with increased risk of fragility fractures independent of estimated GFR. Kidney Int Rep. 2023;8(11):2315–2325. 10.1016/j.ekir.2023.08.016 PMC10658242 38025225

[31] Chu CD, Xia F, Du Y, et al. Estimated prevalence and testing for albuminuria in US adults at risk for chronic kidney disease. JAMA Netw Open. 2023;6(7):e2326230. 10.1001/jamanetworkopen.2023.26230 PMC10375308 37498594

[32] Jørgensen L, Jenssen T, Ahmed L, Bjørnerem A, Joakimsen R, Jacobsen BK. Albuminuria and risk of nonvertebral fractures. Arch Intern Med. 2007;167(13):1379–1385. 10.1001/archinte.167.13.1379 17620531

[33] Schmidt-Gayk H, Grawunder C, Tschöpe W, et al. 25-hydroxy-vitamin-D in nephrotic syndrome. Lancet. 1977;2(8029):105–108. 10.1016/S0140-6736(77)90118-0 69193

[34] Doorenbos CR, de Cuba MM, Vogt L, et al. Antiproteinuric treatment reduces urinary loss of vitamin D-binding protein but does not affect vitamin D status in patients with chronic kidney disease. J Steroid Biochem Mol Biol. 2012;128(1-2):56–61. 10.1016/j.jsbmb.2011.09.002 21958677

[35] Barzilay JI, Peterson D, Cushman M, et al. The relationship of cardiovascular risk factors to microalbuminuria in older adults with or without diabetes mellitus or hypertension: the cardiovascular health study. Am J Kidney Dis. 2004;44(1):25–34. 10.1053/j.ajkd.2004.03.022 15211434

[36] Barzilay JI, Bůžková P, Fink HA, et al. Systemic markers of microvascular disease and bone mineral density in older adults. Osteoporos Int. 2016;27(11):3217–3225. 10.1007/s00198-016-3649-927250972

[37] Aguirre J, Buttery L, O’Shaughnessy M, et al. Endothelial nitric oxide synthase gene-deficient mice demonstrate marked retardation in postnatal bone formation, reduced bone volume, and defects in osteoblast maturation and activity. Am J Pathol. 2001;158(1):247–257. 10.1016/S0002-9440(10)63963-6 PMC1850250 11141498

[38] Griffith JF, Yeung DKW, Tsang PH, et al. Compromised bone marrow perfusion in osteoporosis. J Bone Miner Res. 2008;23(7):1068–1075. 10.1359/jbmr.080233 18302498

[39] Barzilay JI, Buzkova P, Bielinski SJ, et al. The association of microvascular disease and endothelial dysfunction with vertebral trabecular bone mineral density: the MESA study. Osteoporos Int. 2024;35(9):1595–1604. 10.1007/s00198-024-07152-y 38913124

[40] Kim M, Han K, Kwon JK, et al. Bone mineral density and the risk of fracture according to eGFR in postmenopausal women. J Am Soc Nephrol. 2026;37(5):984–994. 10.1681/ASN.0000000960 PMC13143439 41563361

[41] Sidibé A, Auguste D, Desbiens LC, et al. Fracture risk in dialysis and kidney transplanted patients: a systematic review. JBMR Plus. 2019;3(1):45–55. 10.1002/jbm4.10067 PMC6339558 30680363

[42] Ferrari S, Akesson KE, Al-Daghri N, et al. Bone microstructure and TBS in diabetes: what have we learned? A narrative review. Osteoporos Int. 2025;36(7):1115–1128. 10.1007/s00198-025-07495-0 40353870

[43] Li G-F, Zhao P-P, Xiao W-J, Karasik D, Xu Y-J, Zheng H-F. The paradox of bone mineral density and fracture risk in type 2 diabetes. Endocrine. 2024;85(3):1100–1103. 10.1007/s12020-024-03926-w 38922479

[44] Grams ME, Coresh J, Matsushita K, et al. Estimated glomerular filtration rate, albuminuria, and adverse outcomes: an individual-participant data meta-analysis. JAMA. 2023;330(13):1266–1277. 10.1001/jama.2023.17002 PMC10548311 37787795

