# Supplementary material for: Albuminuria, glomerular filtration rate, and fracture among U.S. adults with and without type 2 diabetes
Source: JBMR Plus. 2026 Oct 9;10(11):ziag166. doi: 10.1093/jbmrpl/ziag166 (PMC13695955; doi:10.1093/jbmrpl/ziag166)
Supplement: Supplementary file 1 [file nhanes_albuminuria_supplement_ziag166(1).docx]

**SUPPLEMENTARY TABLES AND FIGURES**

Albuminuria, glomerular filtration rate, and fracture among U.S. adults with and without type 2 diabetes

Simon Hsu, MD, MS,^1^ Leila R. Zelnick, PhD^1^, Ian H. de Boer, MD, MS^1^, Mishaela R. Rubin, MD, MS^2^, Thomas L. Nickolas, MD, MS^3^

^1^Division of Nephrology and Kidney Research Institute, Department of Medicine, University of Washington, Seattle, WA, USA

^2^Metabolic Bone Disease Units, Department of Medicine, Columbia University, New York, NY, USA

^3^Division of Bone and Mineral Diseases, Department of Medicine, Washington University in St. Louis, St. Louis, MO, USA

**Supplementary Table 1.** Associations of albuminuria with hip fracture stratified by type 2 diabetes status and eGFR with additional adjustment for bone mineral density and hs-CRP.

|  |  |  | **OR (95% CI)** | | | |
| --- | --- | --- | --- | --- | --- | --- |
|  | N at risk  (N events) | Weighted prevalence of hip fracture, % (95% CI) | Model 1 | Model 2 | BMD-adjusted | hsCRP-adjusted |
| **Overall** | 20,034 (417) |  | 1.69 (1.25, 2.29)^1^ | 1.59 (1.13, 2.23)^1^ | 1.35 (0.72, 2.53) | 1.72 (1.18, 2.49)^1^ |
| **T2D status** |  |  |  |  |  |  |
| No T2D | 15,431 (334) | 1.9% (1.6%, 2.1%) | 1.93 (1.37, 2.71)^1^ | 1.71 (1.16, 2.50)^1^ | 1.61 (0.76, 3.39) | 1.92 (1.26, 2.93)^1^ |
| T2D | 4,603 (83) | 1.8% (1.3%, 2.4%) | 1.36 (0.80, 2.33) | 1.43 (0.83, 2.47) | 0.80 (0.27, 2.37) | 1.30 (0.74, 2.29) |
| **eGFR** |  |  |  |  |  |  |
| ≥60 | 17,075 (324) | 1.7% (1.4%, 2.0%) | 1.73 (1.22, 2.45)^1^ | 1.64 (1.12, 2.39)^1^ | 1.52 (0.79, 2.91) | 1.72 (1.14, 2.61)^1^ |
| <60 | 2,959 (93) | 3.2% (2.5%, 4.1%) | 1.63 (1.00, 2.64)^1^ | 1.47 (0.87, 2.50) | 0.96 (0.28, 3.30) | 1.68 (0.98, 2.88) |
| **Combined T2D-eGFR** | |  |  |  |  |  |
| No T2D, eGFR ≥60 | 13,459 (267) | 1.7% (1.4%, 2.0%) | 1.94 (1.30, 2.90)^1^ | 1.77 (1.16, 2.72)^1^ | 1.84 (0.87, 3.92) | 1.97 (1.24, 3.15)^1^ |
| No T2D, eGFR <60 | 1,972 (67) | 3.4% (2.5%, 4.5%) | 1.93 (1.09, 3.43)^1^ | 1.52 (0.80, 2.90) | 0.89 (0.19, 4.23) | 1.74 (0.90, 3.36) |
| T2D and eGFR ≥60 | 3,616 (57) | 1.6% (1.0%, 2.3%) | 1.44 (0.71, 2.92) | 1.40 (0.69, 2.85) | 0.78 (0.18, 3.32) | 1.18 (0.56, 2.50) |
| T2D and eGFR <60 | 987 (26) | 2.6% (1.7%, 4.2%) | 1.18 (0.47, 2.96) | 1.56 (0.62, 3.94) | 1.42 (0.17, 11.89) | 1.70 (0.63, 4.55) |

The reference group for each stratum is no albuminuria. The units of eGFR are mL/min per 1.73 m^2^. Model 1 is adjusted for age, age squared, sex, race and ethnicity, and NHANES cycle. Model 2 additionally adjusts for smoking status, history of osteoporosis diagnosis and treatment, history of daily glucocorticoid use, and body mass index. The BMD-adjusted model adjusts for model 2 covariates plus femoral neck BMD and is restricted to participants with available DXA measurements (N = 7,005). The hsCRP-adjusted model adjusts for model 2 covariates plus hsCRP and is restricted to participants with available hsCRP measurements (N = 17,519).

^1^p<0.05

OR, odds ratio; CI, confidence interval; T2D, type 2 diabetes; eGFR, estimated glomerular filtration rate; NHANES, National Health and Nutrition Examination Survey; DXA, dual-energy x-ray absorptiometry; BMD, bone mineral density; hsCRP, high sensitivity C-reactive protein

**Supplementary Table 2.** Associations of albuminuria with any fracture stratified by type 2 diabetes status and eGFR with additional adjustment for bone mineral density and hs-CRP.

|  |  |  | **OR (95% CI)** | | | |
| --- | --- | --- | --- | --- | --- | --- |
|  | N at risk  (N events) | Weighted prevalence of any fracture, % (95% CI) | Model 1 | Model 2 | BMD-adjusted | hsCRP-adjusted |
| **Overall** | 20,034 (2,703) | 15.0% (14.3%, 15.8%) | 1.08 (0.96, 1.22) | 1.03 (0.91, 1.16) | 0.99 (0.79, 1.24) | 1.02 (0.90, 1.16) |
| **T2D status** |  |  |  |  |  |  |
| No T2D | 15,431 (2,140) | 15.1% (14.4%, 15.9%) | 1.14 (0.99, 1.32) | 1.06 (0.91, 1.24) | 0.93 (0.67, 1.29) | 1.06 (0.90, 1.25) |
| T2D | 4,603 (563) | 14.7% (13.1%, 16.3%) | 0.99 (0.77, 1.26) | 0.98 (0.76, 1.27) | 1.18 (0.74, 1.86) | 0.97 (0.73, 1.28) |
| **eGFR** |  |  |  |  |  |  |
| ≥60 | 17,075 (2,265) | 14.8% (14.0%, 15.7%) | 1.15 (1.00, 1.32)^1^ | 1.08 (0.94, 1.25) | 1.01 (0.79, 1.30) | 1.07 (0.92, 1.25) |
| <60 | 2,959 (438) | 16.6% (14.8%, 18.6%) | 0.88 (0.69, 1.14) | 0.85 (0.65, 1.11) | 0.91 (0.55, 1.53) | 0.86 (0.66, 1.12) |
| **Combined T2D-eGFR** |  |  |  |  |  |  |
| No T2D, eGFR ≥60 | 13,459 (1,824) | 14.9% (14.0%, 15.7%) | 1.14 (0.95, 1.36) | 1.07 (0.89, 1.29) | 0.95 (0.67, 1.36) | 1.06 (0.87, 1.30) |
| No T2D, eGFR <60 | 1,972 (316) | 17.5% (15.3%, 19.9%) | 1.13 (0.82, 1.56) | 1.04 (0.73, 1.47) | 0.92 (0.47, 1.80) | 1.03 (0.72, 1.46) |
| T2D and eGFR ≥60 | 3,616 (441) | 14.7% (12.9%, 16.7%) | 1.18 (0.88, 1.57) | 1.14 (0.85, 1.54) | 1.30 (0.76, 2.21) | 1.10 (0.80, 1.51) |
| T2D and eGFR <60 | 987 (122) | 14.6% (11.2%, 18.2%) | 0.57 (0.35, 0.93)^1^ | 0.62 (0.37, 1.04) | 0.70 (0.25, 1.97) | 0.64 (0.37, 1.10) |

The reference group for each stratum is no albuminuria. The units of eGFR are mL/min per 1.73 m^2^. Model 1 is adjusted for age, age squared, sex, race and ethnicity, and NHANES cycle. Model 2 additionally adjusts for smoking status, history of osteoporosis diagnosis and treatment, history of daily glucocorticoid use, and body mass index. The BMD-adjusted model adjusts for model 2 covariates plus femoral neck BMD and is restricted to participants with available DXA measurements (N = 7,005). The hsCRP-adjusted model adjusts for model 2 covariates plus hsCRP and is restricted to participants with available hsCRP measurements (N = 17,519).

^1^p<0.05

OR, odds ratio; CI, confidence interval; T2D, type 2 diabetes; eGFR, estimated glomerular filtration rate; NHANES, National Health and Nutrition Examination Survey; DXA, dual-energy x-ray absorptiometry; bone mineral density; hsCRP, high sensitivity C-reactive protein

**Supplementary Figure 1.** Cohort assembly. NHANES cycles 2011-2012 and 2015-2016 were excluded due to unavailable fracture data.

**Supplementary Figure 2.** Trends in fracture prevalence in NHANES (1999-2020) by albuminuria and eGFR categories. The units of eGFR are mL/min per 1.73 m^2^.

**Supplementary Figure 3.** Trends in fracture prevalence in NHANES (1999-2020) by albuminuria and type 2 diabetes categories.
